# New Insights into Adsorption Properties of the Tubular Au_26_ from AIMD Simulations and Electronic Interactions

**DOI:** 10.3390/molecules28072916

**Published:** 2023-03-24

**Authors:** Ying Meng, Qiman Liu

**Affiliations:** 1 School of Chemistry and Materials Engineering, Huainan Normal University, Huainan 232000, China; 2 Anhui Province Key Laboratory of Low Temperature Co-Fired Materials, Huainan 232000, China

**Keywords:** Au_26_ cluster, adsorption structures, stability, electronic interactions

## Abstract

Recently, we revealed the electronic nature of the tubular Au_26_ based on spherical aromaticity. The peculiar structure of the Au_26_ could be an ideal catalyst model for studying the adsorptions of the Au nanotubes. However, through Google Scholar, we found that no one has reported connections between the structure and reactivity properties of Au_26_. Here, three kinds of molecules are selected to study the fundamental adsorption behaviors that occur on the surface of Au_26_. When one CO molecule is adsorbed on the Au_26_, the σ-hole adsorption structure is quickly identified as belonging to a ground state energy, and it still maintains integrity at a temperature of 500 K, where σ donations and π-back donations take place; however, two CO molecules make the structure of Au_26_ appear with distortions or collapse. When one H_2_ is adsorbed on the Au_26_, the H–H bond length is slightly elongated due to charge transfers to the anti-bonding σ* orbital of H_2_. The Au_26_-H_2_ can maintain integrity within 100 fs at 300 K and the H_2_ molecule starts moving away from the Au_26_ after 200 fs. Moreover, the Au_26_ can act as a Lewis base to stabilize the electron-deficient BH_3_ molecule, and frontier molecular orbitals overlap between the Au_26_ and BH_3_.

## 1. Introduction

Gold clusters have attracted intensive research interest in recent decades due to their mystical properties and appealing structural beauty, giving rise to promising applications in catalysis, chemo-sensing, optical materials, and energy conversion [1,2,3,4,5,6]. Particular attention has been focused on understanding and expanding functional properties, requiring the characterization of well-defined clusters with atomic precision [7,8,9,10,11]. The structures of Au clusters and their growth patterns have been well characterized, in which small-sized clusters exhibit a wide range of planar structures compared with Cu and Ag clusters, while medium-sized clusters have more exotic structures, such as the pyramidal Au_20_ and icosahedral Au_32_ cages [12,13,14]. These unusual geometric structures are traced to the strong relativistic effect and aurophilic attraction of gold. Especially interesting, the Au_20_ pyramidal cluster is an ideal model of a catalyst and building blocks for assembled materials because of its highly symmetric tetrahedral structure and large HOMO−LUMO gap of 1.77 eV [12]. Furthermore, the relation between the stability that is quantified either in terms of the binding energy per atom or in terms of dissociation energies and the electronic configurations of Au clusters is also well established: the Jellium model and spherical aromaticity rule are applicable to compact structures and cage structures, respectively, and the SVB theory is applicable to prolate clusters [15,16].

Since Haruta et al. discovered that small-sized Au clusters can be capable of catalyzing CO oxidation below room temperature, Au clusters are used as efficient catalysts in various important chemical transformations, even though bulk Au is one of the most chemically inert metals in the periodic table of elements [17,18,19]. For example, Zielasek and co-workers reported applications of nano-gold catalysts in gas masks for the oxidization of toxic chemicals, in bathrooms for the removal of odor compounds, or in vehicles for the conversion of CO to CO_2_ [20]. Wu et al. investigated the electronic structure, optical properties, and PESs of the H_2_-Au_6_ model system, in order to explore key pathways in LSPR-promoted chemical reactions [21]. Yan found that the rate of water splitting on the Au nanoparticles is dependent not only on respective optical absorption strength but also on the quantum oscillation mode of plasmonic excitation [22]. Zhu and co-workers used the Au_10_ cluster as an adsorption model and found that the cluster can be highly active for CO oxidation both in the gas phase and on the rectangular TiO_2_ support [23]. Lee and Kim reported that a small anionic Au_6_ is effective in the activation of CO_2_ and show that such characteristics result from orbital interactions between the HOMO of Au_6_^−^ and the LUMO of CO_2_ [24]. Sengupta and Chung unveiled reaction mechanisms of the hydrogenation of ethylene on two popular magic Au_8_ and Au_20_ [25]. Recently, we constructed a series of isoelectronic pyramidal clusters based on the framework of the Au_20_ cluster and analyzed their adsorption properties and electron structures for CO molecules, which develops a new way to extend superatom catalysts from superatomic clusters and also gives an inference for extensions of single-atom catalysts [26]. 

Among Au clusters with unusual structures, the Au_26_ belongs to one of the special species. Initially, Dong et al. showed that a tubular structure with high *D*_6_d symmetry is a possible ground state in the energy which is considered as a combination of four Au_6_ rings and two extra Au atoms at the center of either side [27]. Later, Wang and coworkers performed an extensive structure search and confirmed that the Au_26_ cluster has a great many metastable isomers within a narrow energy window and is a typical fluxional system [28]. Based on this, Joshi et al. found that the tubular *D*_6_d structure has the most thermal stability among the three compact and core-shell structures and one tubular structure [29]. Recently, we reanalyzed this system and revealed that the electronic nature of the large HOMO-LUMO energy gap (1.34 eV) of tubular Au_26_ was due to that the tubular structure can be viewed as a superatomic molecule consisting of two open-shell Au_13_ moieties that achieve shell closure via a super triple bond (σ, 2π) based on spherical aromaticity [30]. 

The peculiar geometric structure of the Au_26_ could be the embryo of a Au nanotube and is an ideal catalyst model for studying the adsorption properties of the Au nanotube. However, we retrieved most of the relevant literature through Google Scholar and did not find that anyone has reported a connection between the geometry structure and reactivity properties of the tubular cluster. Here, we selected electron donors CO molecules and H atoms, H_2_ molecules, and the electron-deficient BH_3_ molecules to study the fundamental adsorption behaviors that occur on the surface of tubular Au_26_ clusters and obtained different types of adsorption structures. The thermal stability, adsorption energies, stretching frequencies, and electronic properties of their adsorption structures are analyzed in detail.

## 2. Results and Discussion

### 2.1. Adsorption Properties of the Tubular Au_26_ Cluster with CO Molecules

The optimized structure of the tubular Au_26_ cluster is determined at PBE0/Def2-TZVP level, and the structure parameters (*D*_6_d symmetry) agree with previous works [27,30]. Its empty structure has four prominent layers. The middle two layers can be seen as the hexatomic ring units without a center Au atom, and the remaining part comprises the top and bottom layers of the nanotube. Except for the unusual geometry, 20 valence electrons of the Au_26_ occupied 5 bonding orbitals (σ*_s_*, 2π*_px_*_,*y*_, 2δ*_dxy_*_,*x*2-*y*2_) and 5 anti-bonding orbitals (σ*_s_, 2π*_px__,*y*_, 2δ*_dxy__,*x*2-*y*2_). The remaining six electrons filled one σ and 2π super bonding orbitals that achieve the electronic shell closure. The detailed analysis of MOs can also be referred to in the previous literature [30]. 

The adsorptions are the basic behaviors of the interfaces, which impact the atomic structures, electronic properties, and catalysis properties of the interfaces. Chemisorption processes are not only related to electrostatic interaction energy but also to the symmetry of frontier orbitals and the matching of energy levels. However, there are too many possibilities for adsorption sites on the medium-sized Au_26_ cluster. Computing molecular electrostatic potential *V*(r) is a well-built strategy to analyze intermolecular interactions and surface charge distributions of the clusters [31]. The *V*(r) results of the Au_26_ cluster showed that six Au atoms in a side face of the tubular structure possess positive charges with six apparent σ-hole areas, respectively, while the negative charges are in the intermediate region of the tubular structure, indicating that Au atoms at both ends of the tubular structure are active sites for both charge-controlled and frontier-controlled interactions with electron donors, e.g., CO and N_2_ molecules. 

As mentioned in the paragraphs of the Introduction, the earlier reports that small-sized Au clusters exhibit a fantastic activity for CO oxidation reactions—revealing mechanisms of catalytic/adsorption behaviors of CO molecules on surfaces of Au clusters—were received with high-level concerns [7,11,32]. It is well-known that the presence of low-coordinated metal atoms on the surfaces of Au-based clusters play a dominant role in enhancing catalytic activity [33,34,35,36,37]. Moreover, in most cases, CO molecules prefer to be top-coordinated on the surfaces of the Au clusters, which are generally explained in terms of σ donations of electron density from CO molecules to the metals and π-back donations from metals to CO [34,36,37,38,39]. Among these adsorption structures, the top-coordinated patterns can make stretching frequencies of CO molecules appear with varying degrees of redshifts.

**Figure 1:**
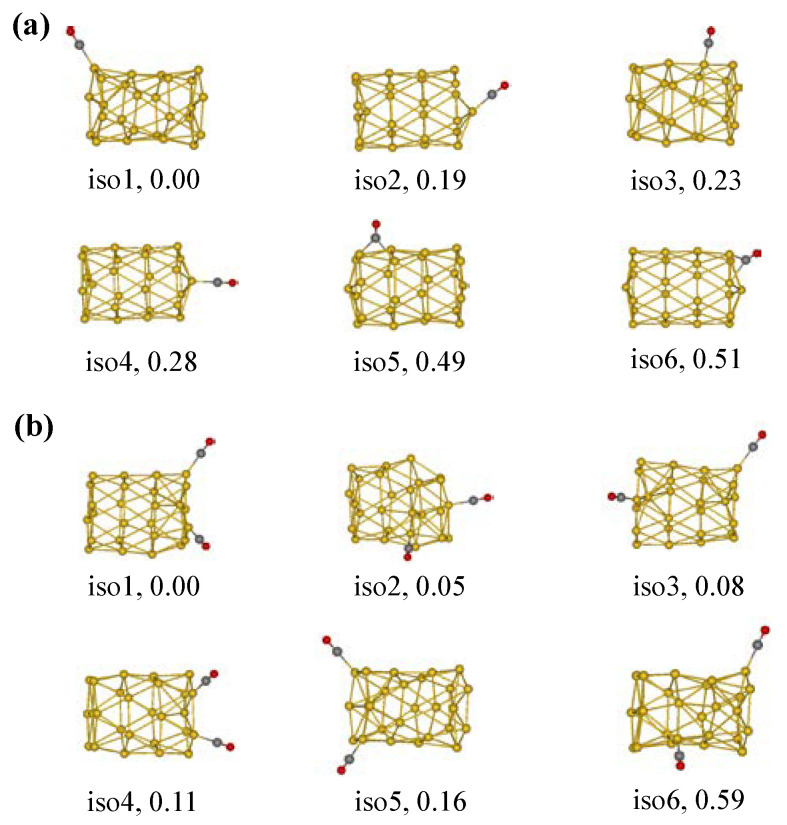
Isomer structures and relative stability of (**a**) Au_26_-CO and (**b**) Au_26_-(CO)_2_. Au, yellow; C, grey; O, red (supplementary materials).

We considered more adsorption sites to evaluate the effectiveness of using σ holes to determine the optimal adsorption sites involving abundant patterns of top sites. Many hollow and bridge sites are also considered for the initial adsorption structures of the Au_26_-CO cluster. In this process, a CO molecule is randomly placed in different positions on the surface of the Au_26_, so that more possible adsorption patterns can be obtained. However, many initial structures after optimizations either have imaginary frequencies or fail to converge to local minimums. It is well known that PBE0 calculations cannot provide a correct description of the nonlocal long-range vdW interactions, which is crucial for improving the description of the weak binding systems. Thus, we employed the D3bj correction proposed by Grimme to improve the description of the Au_26_–CO interactions [40]. Figure 1a shows the isomer structures of the Au_26_ cluster with one CO molecule, only the six stable ones. Among these isomers, the σ-hole adsorption structure (corresponding to iso1) is quickly identified as belonging to a true local minimum, and it has a ground state energy. At this time, the structural framework of the Au_26_ nanotube in the iso1 hardly undergoes any deformation. Furthermore, iso1 is energetically more preferable because the difference in energy between iso1 and other adsorption isomers is very pronounced. In comparison, the Au_26_ nanotube structures in other isomers collapse to varying degrees, which also further confirms that σ-hole regions are the best adsorption sites.

**Figure 2:**
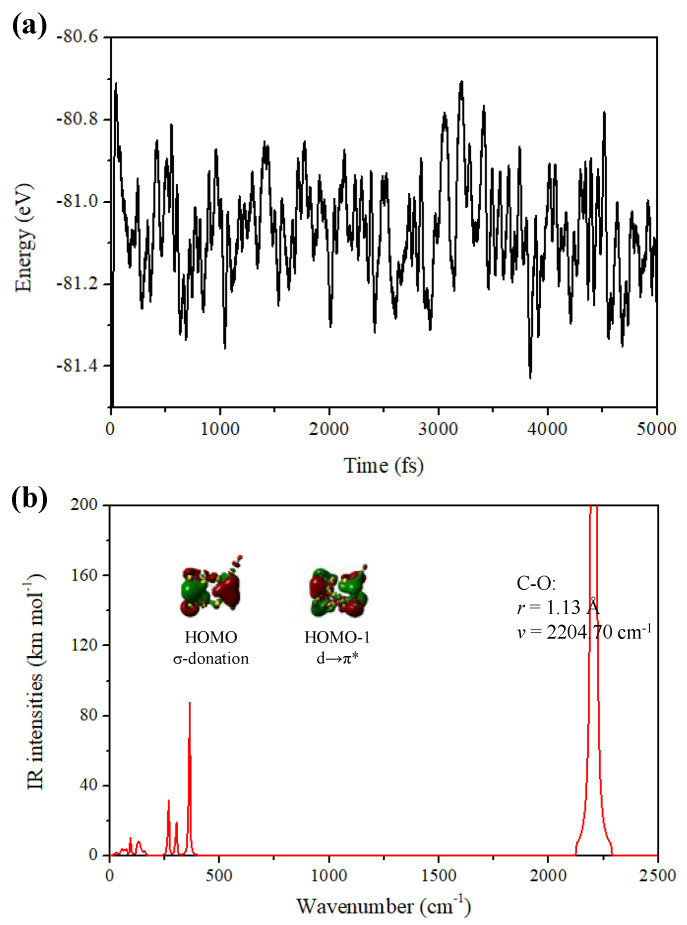
(**a**) The energy fluctuation with the time step at 500 K, (**b**) infrared spectrum and molecular orbitals of iso1 structure of the Au_26_-CO.

To further study the thermal stability of the iso1 structure in Figure 1a, we carry out the ab initio molecular dynamics (AIMD) simulations in the VASP software package [41]. In the modeling process, a 20 × 20 × 20 Å box for the initial structure is built to avoid interaction between the individual clusters. Temperatures 300 K and 500 K are selected as the initial temperatures of the simulations. The simulation time of each temperature lasts 10 ps, and the time step sets 2.0 fs. During the simulations, snapshots are extracted every 20 fs to observe the details of structural changes, and the snapshots are plotted after 10 ps AIMD simulations. The energy fluctuation of AIMD simulations with the time steps is plotted in Figure 2a. It is found that the structure of Au_26_-CO with the σ-hole adsorption still maintains integrity at a temperature of 500 K, in which there are only slight disturbances of individual atoms, suggesting its good thermal stability in a high-temperature environment. 

Earlier findings have also confirmed that CO molecules have propensities to adsorb at low-coordinated gold atoms (top sites) of Au-based clusters, which can be treated by the Blyholder model that the σ donations and π-back donations take place, resulting in the CO stretching frequencies have evident redshifts. Based on this, the result expressed in the above paragraph is in line with our expectations. Figure 2b shows the molecular orbitals and infrared spectrum (IR) of the most stable iso1 of the adsorption structures of the Au_26_-CO. Although several other local minima have been given, here we focus on the most stable minima structure wherein the CO as a Lewis base is close to the σ-hole region. Accompanying the chemisorption of CO, the Au–C distance is 1.97 Å and it conforms to the range of reported Au–C bonds (1.98–2.00 Å), where the major interaction belongs to the σ-donation (HOMO orbital) interaction from the 5σ orbital of a CO molecule to empty orbitals of the cluster. The C=O bond distance of 1.13 Å slightly elongates due to the electron transfer to the anti-bond π orbital, as shown in the HOMO-1 orbital of Au_26_-CO. Moreover, the C–O frequency (2204.70 cm^−1^) of this structure is <free CO (2239.87 cm^−1^) [42,43]. Compared to other Au-based clusters, the red-shift of the C–O bond is not obvious, and due to that the Au_26_ cluster is a cage–cage superatomic molecule based on spherical aromaticity and kept chemically inert in the geometrical and electronic structure. 

For further research, more CO molecules were selected to simultaneously adsorb on the surface of the Au_26_ cluster. We also considered various adsorption situations during the construction of the initial configurations. Optimized isomer structures of the Au_26_ cluster with two CO molecules are shown in Figure 1b. Unfortunately, it was found that the adsorptions of two CO molecules generate major changes in the parent geometry of the Au_26_ cluster. Among them, tubular walls of adsorption substrates of most isomers have different degrees of collapse or distortion. For iso5, the tubular structure is maintained, and its stability is related to electrostatic interactions. In brief, for the CO electron donors, the active sites of the Au_26_ cluster only exist in σ-hole regions and its geometric structure is prone to deformations when CO fragments adsorb at other positions. Therefore, this cluster is not suitable as a potential catalyst for the C–O bond activation.

### 2.2. Adsorption Properties of the Tubular Au_26_ Clusters with Hydrogens

Hydrogen atoms are present in most of the materials on earth. The understanding of the chemical bonding of H atoms with other elements became a fundamental problem in chemistry, biology, and physics [44,45,46,47]. Of particular interest currently are hydride coinage metal clusters that are combinations of metals/hydrogens/electrons, wherein the hydrides are embedded in or ligated to the metal frameworks [48,49,50]. Moreover, it is worth mentioning that the interaction between hydrogen atoms and clusters is an important issue that needs to be further explored in heterogeneous catalysis. For instance, Tsukuda et al. found that 1.2-nm gold clusters can adsorb hydrogen on the surfaces and become plasmonic, and this phenomenon was attributed to the electron doping of Au sp bands by adsorbed hydrogen atoms, which has been demonstrated by direct spectroscopic evidence for the H-mediated modulation [51,52]. Subsequently, they further showed that hydrogen atoms also donate 1*s* electrons when they are bonded to other gold clusters, in which the hydrogens typically act as doped metal atoms. Jiang et al. showed whether hydrogen atoms withdraw or donate electrons in gold clusters depends on the ligands, the local environments, and the electron counts [53].

**Figure 3:**
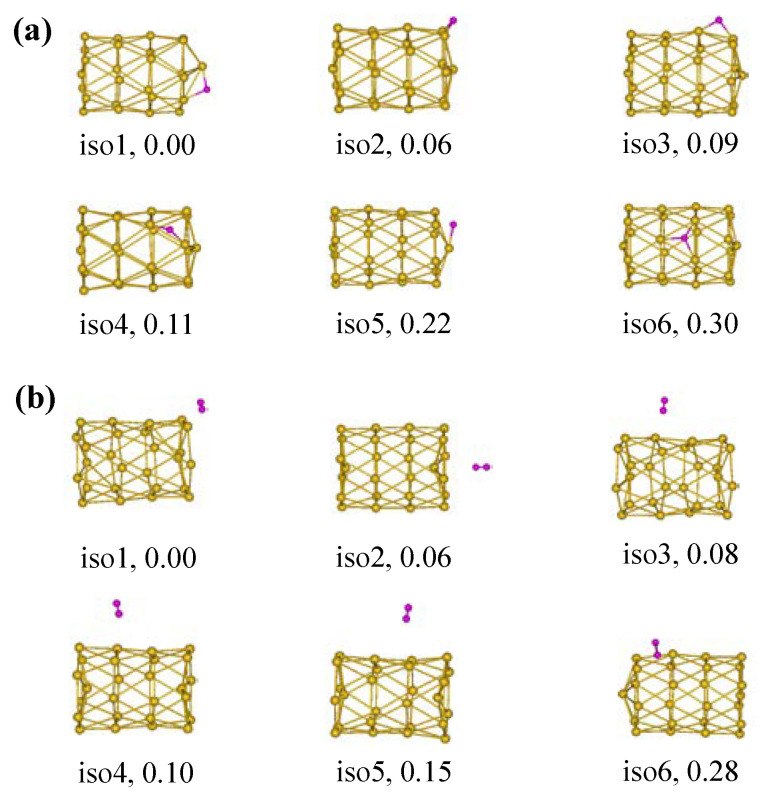
Isomer structures and relative stability of (**a**) Au_26_-H and (**b**) Au_26_-H_2_. Au, yellow; H, pink.

Studies of the interactions between hydrogen and Au clusters are essential to unveil the origins of unusual chemical activities of Au, which are currently the most extensively studied subjects in heterogeneous catalysis and surface chemistry. Hence, the interactions between cluster Au_26_ and atomic H are also investigated in the present work. The possible structures of Au_26_–H_2_ complexes are explored. Figure 3a shows the isomer structures of the Au_26_ cluster with one H atom at the PBE0/Def2-TZVP level, according to the sequence of their stability. Unfortunately, no matter where the H atom is located on the surface of the Au_26_ cluster, it was found that the atomic H adsorptions generate major changes in the initial parent geometry, in which we can clearly see that tubular walls of adsorption substrates of all given isomers have different degrees of distortions or collapse. It can be said, even though the H atom is very small, the integrity of the electronic structure of the Au_26_ cluster is also easily destroyed because the hydrogen atom withdraws or donates electrons.

To further exclude the influence of electronic effects, we selected one H_2_ molecule to be adsorbed on the surface of the Au_26_ cluster. In fact, as a simple model system for heterogeneous catalysis, the interactions and reactive dynamics of H_2_ on surfaces of Au-based clusters have been studied extensively under well-defined surface science conditions. However, given the 4.5 eV bond energy of the H_2_ molecule, the activation of the H–H bond is often a difficult step. Moreover, molecular adsorption is a complex mechanism. In this study, one H_2_ molecule was randomly placed at different sites on the surface of the tubular framework. Figure 3b shows the isomer structures of the Au_26_ cluster with one H_2_ molecule, involving the top, hollow, and bridge adsorption structures. The preferred adsorption configuration (corresponding to iso1) is the H_2_ molecule on top of a Au atom. Such a pattern has often been observed in other alloy clusters with H_2_. It is favorable for H_2_ in the form of molecules to adsorb on the Au_26_, resulting in the unbroken H–H bond. The integrity of the tubular structures can be maintained without collapse, though H_2_ molecules adsorb to different positions of these isomers. 

**Figure 4:**
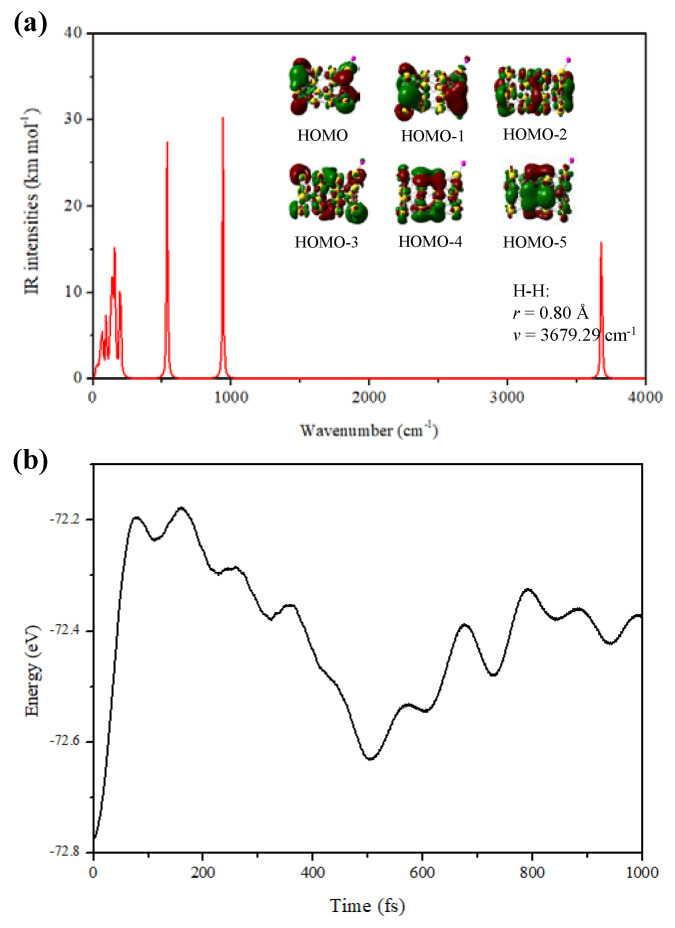
(**a**) Infrared spectrum and molecular orbitals, (**b**) the energy fluctuation with the time step at 300 K of iso1 structure of the Au_26_-H_2_.

The equilibrium bond length of a free H_2_ molecule is computed to be 0.74 Å at the PBE0/Def2-TZVP level, which is in good agreement with the experimental value. Figure 4a shows the molecular orbitals and IR spectrum of the most stable iso1 of the Au_26_-H_2_. The stretching frequency of the H–H bond shows a red-shifting phenomenon, and the bond length (0.795 Å) is slightly elongated, longer than that of a free H_2_. The fundamental role of vibrational spectra in the detection of structural changes in atomic clusters is well known and, hence, it is needed to recognize the minimum and maximum values of frequencies under conditions where these changes are expected, e.g., due to interactions with one or several H_2_ molecules. The HOMO-1 and HOMO-3 orbital charge density shown in Figure 4a confirms an overlap between the frontier orbitals of the cluster and the H_2_ molecule, indicating that charge transfers from the Au_26_ to the antibonding σ* orbital of H_2_ occurred. Moreover, the electronic properties of Au_26_ also change upon H_2_ adsorption. For instance, the HOMO-LUMO gap (1.63 eV) of the iso1 structure is much broader, 0.29 eV, larger than that of a free Au_26_, showing that H_2_ is not physically adsorbed on the cluster. Because of this large HOMO-LUMO gap, the interaction of H_2_ with Au_26_ is weak (molecular adsorption is generally weak), with an adsorption energy of only 0.26 eV, and low reactivity is indeed expected.

To explore the adsorption and desorption processes at room temperature, we also carried out an AIMD simulation of the Au_26_-H_2_ for 1000 fs. It is observed in Figure 4b that the iso1 adsorption structure of Au_26_-H_2_ can maintain integrity within 100 fs at 300 K with only slight disturbances of individual atoms, and the H_2_ molecule started moving away from the Au_26_ cluster after 200 fs. Moreover, the H_2_ molecule absorbed in the molecular form with negligible deformation in the cluster during simulations. In brief, no significant changes are found in the geometry parameters of the cluster even after desorption. Thus, the Au_26_ cluster has the potential to use for reversible H_2_ storage.

### 2.3. Adsorption Properties of the Tubular Au_26_ Clusters with a BH_3_ Molecule

The borane molecule, BH_3_, is one of the simplest and smallest examples (other than molecular hydrogen) of an electronically neutral bonded species with an incomplete octet. However, experimental detection of BH_3_ was long hindered by its high reactivity. The earliest spectroscopic observation of BH_3_ was achieved by Kaldor and Porter, which is able to be produced by BH_3_CO pyrolysis [54]. The BH_3_CO molecule has a complete octet, or the CO molecule gives two electrons to BH_3_ to stabilize it. In fact, the BH_3_ molecule normally acts as a Lewis acid, in which the empty orbitals of central atoms of the Lewis acids accept pairs of electrons from Lewis bases. For example, ammonia borane (NH_3_BH_3_) is the result of a Lewis acid–base interaction, which is considered to be one of the most promising hydrogen storage materials because of its high hydrogen-content capacity [55]. Recently, Bowen et al. reported a (Na-BH_3_)^−^ cluster, featuring a non-trivial Na→BH_3_ dative bond, representing an example of a Lewis adduct with an alkalide as the base [56]. 

**Figure 5:**
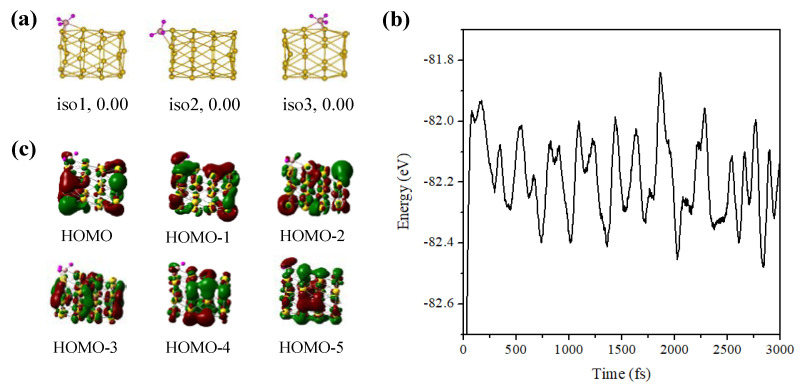
(**a**) Isomer structures and relative stability of the Au_26_-BH_3_, (**b**) molecular orbitals, and (**c**) the energy fluctuation with the time step at 500 K of iso1 structure of Au_26_-BH_3_. Au, yellow; H, pink; N, brown.

Figure 5a shows the isomer structures of the Au_26_ cluster with one BH_3_ molecule. The many initial adsorption structures after structural optimizations only converge to the three stable ones, including two hollow adsorptions (iso1 and iso3) and one bridge adsorption structure. It is found that the tubular structure of the Au_26_ cluster was only slightly changed when the BH_3_ molecule is adsorbed to different positions but the tubular walls of adsorption substrates of all given isomers did not collapse. Furthermore, the AIMD simulation in Figure 5b shows that the Au_26_-BH_3_ structure still has good thermal stability at 500 K. In order to examine chemical interactions between fragments, we calculated the binding energy between the BH_3_ molecule and the Au_26_ cluster. The binding energy of 2.58 eV is in the category of covalent bonds. Moreover, the HOMO and HOMO-1 orbitals from Figure 5c show that there are overlaps between the frontier orbitals of the cluster and the BH_3_ molecule. Hence, the Au_26_ cluster can act as a Lewis base to stabilize the electron-deficient BH_3_ molecule. 

## 3. Computational Methods and Details

The structure optimizations of adsorption clusters and subsequent computations are performed using the PBE0 functional with the dispersion correction (D3bj) and Def2-TZVP basis set [57,58,59]. The basis set is selected to consider the relativistic effective core potential for heavy Au atoms. Energies of structure isomers of adsorption clusters reported herein are considered contributions of the zero point energy (ZPE) correction. It is confirmed that the isomer structures of each component belong to true local minimum points by analyzing the vibration frequency at the same theoretical level. The quality of self-consistent field (SCF) convergence tolerance is set with a convergence criterion of 1 × 10^−6^ hartree on total energy and electron density, 2 × 10^−3^ hartree Å^−1^ on the gradient, and 5 × 10^−3^ Å on the displacement in our calculations. All first-principles calculations are based on the Gaussian 09 package [60]. The visualization of the molecular orbitals (MOs) is achieved in the MOLEKEL 5.4 program [61]. 

The ab initio molecular dynamics (AIMD) simulations are implemented in the Vienna ab initio simulation (VASP) package [62]. The ion–electron interaction was described using the projector-augmented plane wave approach. The generalized gradient approximation expressed by PBE functional and a 450 eV cutoff for the plane-wave basis set were adopted in the computations. The convergence threshold was set as 10^−4^ eV in energy and 0.01 eV Å^−1^ in force. The AIMD temperatures are controlled by using the Nose’–Hoover method [63].

## 4. Conclusions

In summary, we investigated the adsorption properties of the tubular Au_26_ from the AIMD simulations and electronic interactions using DFT methods. Three kinds of molecules with different electronic structures were selected to analyze the fundamental adsorption behaviors that occur on the surface of tubular Au_26_ clusters. When the electron donor CO molecules are adsorbed on the Au_26_, the σ-hole adsorption structure with one CO molecule is quickly identified as belonging to a ground state energy, and it maintains integrity at a temperature of 500 K, where the C=O bond distance of 1.13 Å is slightly elongated due to the electron transfer to the anti-bond π orbital. However, two CO molecules make the structure of Au_26_ appear with distortions or collapse. When one H_2_ molecule is adsorbed on the Au_26_, the preferred configuration is the H_2_ molecule on top of a Au atom, in which the H–H stretching frequency has red-shifting and the bond length is slightly elongated because of charge transfers to the antibonding σ* orbital of H_2_. The adsorption structure of Au_26_-H_2_ can maintain integrity within 100 fs at 300 K, and the H_2_ molecule started moving away from the Au_26_ cluster after 200 fs. However, the H atom makes the structure of Au_26_ appear with distortions or collapse. The isomer structures of the Au_26_ cluster with one BH_3_ molecule included hollow adsorption and bridge adsorption structures, and their frontier molecular orbitals have overlaps between the Au_26_ and BH_3_.

For CO electron donors, the active sites of the Au_26_ cluster only exist in σ-hole regions, and its geometric structure is prone to deformations when the fragments adsorb at other positions. The H_2_ molecule adsorbs to different positions of the Au_26_, and the integrity of the tubular structure can be maintained without collapse. Moreover, the Au_26_ cluster can act as a Lewis base to stabilize the electron-deficient BH_3_ molecule.

## Data Availability

The data presented in this study are available upon request from the corresponding authors.

## Supplementary Materials

The following supporting information can be downloaded at: https://www.mdpi.com/article/10.3390/molecules28072916/s1, The calculated energies and cartesian coordinates of the Au_26_-CO, Au_26_-(CO)_2_, Au_26_-H, Au_26_-H_2_, Au_26_-NH_3_.
