# Supplementary material for: New Insights into Adsorption Properties of the Tubular Au26 from AIMD Simulations and Electronic Interactions
Source: Molecules. 2023 Mar 24;28(7):2916. doi: 10.3390/molecules28072916 (PMC10096096; doi:10.3390/molecules28072916)
Supplement: Supplementary file 1 [file molecules-28-02916-s001.zip › molecules-2287587-supplementary.pdf]

Supporting Information

New insights into adsorption properties of the tubular Au_{26} from the AIMD simulations and electronic interactions

Ying Meng[1], Qiman Liu[1, 2,*]

[1]School of Chemistry and Materials Engineering, Huainan Normal University, Huainan 232000, P. R. China

[2]Anhui Province Key Laboratory of Low Temperature Co-fired Materials, Huainan 232000, P. R. China.

***Corresponding author.** E-mail: qimliu@ustc.edu.cn

The calculated energies and cartesian coordinates of iso1 of the Au_{26}-CO

28

E= -99150.410799 eV E_{HL} = 1.568 eV

Au	3.669258	-1.402727	-2.238521
Au	3.572335	-2.879354	0.124313
Au	3.456111	-1.408152	2.474599
Au	3.456205	1.404620	2.476283
Au	3.572605	2.878943	0.128046
Au	3.669396	1.405476	-2.236730
Au	1.267894	-2.616157	-1.367587
Au	1.137620	-2.636298	1.418304
Au	1.084074	-0.001740	2.341740
Au	1.137801	2.634077	1.421472
Au	1.268286	2.618180	-1.364567
Au	1.290154	0.001523	-2.280796
Au	3.217310	-0.000232	0.105069
Au	-1.047985	-1.375150	-2.168664
Au	-1.133032	-3.263237	-0.118912
Au	-1.230862	-1.367409	1.903869
Au	-1.230493	1.363835	1.903320
Au	-1.132569	3.263882	-0.115388
Au	-1.047733	1.378649	-2.167902
Au	-3.446146	-2.518013	-1.598648
Au	-3.566059	-2.529628	1.163157
Au	-3.640448	-0.000932	2.578435
Au	-3.565523	2.528789	1.166032
Au	-3.445903	2.520093	-1.595656
Au	-3.436482	0.001798	-2.745127
Au	-2.974925	-0.000020	-0.049182
C	-4.721336	-0.003398	4.224912
O	-5.355277	-0.005477	5.156355

The calculated energies and cartesian coordinates of iso1 of the Au_{26}-$(CO)_2$

30

E= -102232.605401 eV E_{HL} = 1.337 eV

Au	-3.432828	-0.762203	2.735263
Au	-3.224692	-2.966984	1.093923
Au	-3.421817	-2.429990	-1.662216
Au	-3.629582	0.176481	-2.658027
Au	-3.862402	2.388312	-1.016795
Au	-3.835102	1.818408	1.744246

Au	-0.904905	-1.911008	2.148005
Au	-0.925292	-3.017769	-0.401265
Au	-1.148760	-0.958773	-2.244762
Au	-1.397670	1.828425	-2.136583
Au	-1.576217	2.904268	0.417368
Au	-1.261747	0.866680	2.247775
Au	-3.382463	-0.273808	0.035859
Au	1.217729	-0.194026	2.096969
Au	1.504708	-2.719345	1.003817
Au	1.258427	-2.141695	-1.838851
Au	1.045864	0.617719	-2.011679
Au	0.778097	3.210761	-1.077624
Au	0.846042	2.498466	1.654428
Au	3.659474	-1.399512	2.472303
Au	3.624338	-3.084877	-0.910168
Au	3.591693	-0.520287	-2.673961
Au	3.288291	1.898793	-1.225995
Au	2.959827	3.901620	0.586769
Au	3.436698	1.448358	1.815554
Au	3.048405	-0.581401	-0.048701
C	4.339512	-0.807418	-4.472142
O	4.789781	-0.946047	-5.495863
C	4.846675	-2.212140	3.804331
O	5.541433	-2.680846	4.558432

The calculated energies and cartesian coordinates of iso1 of the Au_{26}-H

27

E= -96084.142134 eV E_{HL} = 1.704 eV

Au	-3.760861	-2.072286	1.525809
Au	-3.745854	-2.294324	-1.236070
Au	-3.483763	-0.079438	-2.876434
Au	-3.449869	2.395662	-1.576441
Au	-3.488296	2.591615	1.174660
Au	-3.539258	0.355278	2.811479
Au	-1.411115	-2.746957	0.210841
Au	-1.244668	-1.557157	-2.300954
Au	-1.079027	1.211633	-2.525428
Au	-1.083293	2.720358	-0.176291
Au	-1.112584	1.616274	2.375683
Au	-1.257418	-1.150718	2.478514
Au	-3.791828	0.159842	-0.029777
Au	0.975073	-2.423392	1.568193
Au	0.952979	-2.703840	-1.209328
Au	1.210035	-0.316502	-2.594947

Au	1.247997	2.257140	-1.545837
Au	1.225568	2.491815	1.229610
Au	1.199100	0.091820	2.649698
Au	3.114560	-3.610350	0.262893
Au	3.416388	-1.505077	-1.457066
Au	3.602495	1.065119	-2.370882
Au	3.585853	2.878637	-0.210134
Au	3.531293	1.455297	2.217397
Au	3.447750	-1.245026	1.635244
Au	4.873662	0.425466	-0.043601
H	5.141320	-0.860094	1.040436

The calculated energies and cartesian coordinates of iso1 of the Au_{26}-H_2

28

E= -96100.227907 eV E_{HL} = 1.632 eV

Au	-3.517230	-1.405544	2.381606
Au	-3.536578	-2.884722	0.031306
Au	-3.567108	-1.407674	-2.329142
Au	-3.566951	1.402765	-2.332060
Au	-3.536549	2.884884	0.025181
Au	-3.517128	1.410753	2.378802
Au	-1.155504	-2.620942	1.403759
Au	-1.187870	-2.617663	-1.386907
Au	-1.187464	-0.002743	-2.310254
Au	-1.187697	2.614138	-1.392193
Au	-1.155654	2.624258	1.398557
Au	-1.141634	0.002555	2.330649
Au	-3.195006	0.000162	0.022116
Au	1.190631	-1.370789	2.048773
Au	1.172208	-3.213420	-0.031492
Au	1.146944	-1.382162	-2.131006
Au	1.147157	1.375750	-2.130965
Au	1.172014	3.213611	-0.037718
Au	1.190507	1.376090	2.047397
Au	3.553893	-2.511659	1.339947
Au	3.523231	-2.508589	-1.428894
Au	3.548186	-0.003002	-2.629307
Au	3.523494	2.504977	-1.433309
Au	3.553413	2.515425	1.335711
Au	3.594177	0.002967	2.674310
Au	3.023521	0.000461	0.044536
H	4.463620	0.402030	4.368184
H	4.463258	-0.393127	4.369226

The calculated energies and cartesian coordinates of iso1 of the Au_{26}-NH_3

30

E= -96792.541877 eV E_{HL} = 1.620 eV

Au	3.529274	2.002593	1.973237
Au	3.492286	2.840112	-0.678071
Au	3.550824	0.852563	-2.609000
Au	3.610075	-1.888811	-1.949877
Au	3.577367	-2.721187	0.698982
Au	3.537786	-0.738333	2.628503
Au	1.123670	2.927368	0.763651
Au	1.116750	2.122822	-1.875583
Au	1.199969	-0.608524	-2.269179
Au	1.256509	-2.944187	-0.779033
Au	1.186237	-2.139433	1.912204
Au	1.152562	0.614499	2.261514
Au	3.277013	0.064466	0.006055
Au	-1.197582	1.890132	1.783206
Au	-1.233640	3.167787	-0.747232
Au	-1.167127	0.664510	-2.057230
Au	-1.080159	-2.132288	-1.995080
Au	-1.111937	-2.985949	0.649673
Au	-1.171452	-0.818967	2.344100
Au	-3.600393	2.795138	0.934108
Au	-3.602705	1.898429	-1.975902
Au	-3.515820	-0.807489	-2.495444
Au	-3.411357	-3.056889	-0.871401
Au	-3.534509	-2.093539	1.758360
Au	-3.563307	0.631659	2.651488
Au	-3.061920	0.049376	0.045503
B	-3.455556	3.963095	-1.014667
H	-4.239019	4.358335	-0.120369
H	-2.620246	4.814894	-1.271107
H	-4.177674	3.728475	-2.031553
